# Occupational Accidents in Iran: Risk Factors and Long Term Trend (2007–2016)

**Published:** 2019-06-22

**Authors:** Nazanin Izadi, Omid Aminian, Bahador Esmaeili

**Affiliations:** ^1^Center for Research on Occupational Disease, Tehran University of Medical Sciences, Tehran, Iran

**Keywords:** Occupational Accident, Injury, Worker, Epidemiology

## Abstract

**Background:** Although much is known about the distribution of occupational accidents in the world, less is known about occupational injuries in developing countries. Therefore, the aim of this study was to investigate the trend of occupational accidents during 10 years (2007-2016) and to find factors affecting the accident outcome.

**Study design:** A cross-sectional study.

**Methods:** This study was done based on the data gathered by the Iranian Social Security Organization (ISSO), including demographic data (age, sex, marital status) and occupational accident characteristics (accident sector, cause, type, body part, location, time, month, and incident outcome).

**Result:** A decreasing pattern of occupational accidents was found from 2.95 per 1000 workers in 2007 to 1.46 per 1000 workers in 2016. The mean age of injured workers was 32.97 years. The most common cause and type of accidents were incaution and lack of attention and collision, and trapping, respectively. Limbs were the most affected body parts, and less than 1% of occupational accidents resulted in death. The highest incident was seen in the industrial sector during all years. More severe accident outcomes were seen at older ages and in the male gender, married subjects, winter season, agriculture sector, and outside of work place.

**Conclusion:** These results provide a basis for further investigations regarding data collection and accident causes. Modification of some associated factors and implementation of safety prevention programs would be useful in reducing occupational accidents in Iran.

## Introduction

Occupational accidents are defined as uncontrolled, undesirable and unplanned events that cause or have the potential to cause unintended damage or serious injury to the body in the workplace. Occupational accidents have been increasing in the developing countries despite improvements in occupational safety standards^1^. Also, adequate information about occupational accidents is not usually available from all countries in the world^2^. According to the International labor organization (ILO), approximately 2.78 million fatal occupational accidents or diseases occur annually. Also, it is estimated that non-fatal work-related injuries and illnesses are around 374 million each year, which result to absences from work. Occupational accidents impose direct, indirect, and hidden costs on the society. The economic burden of occupational accidents exceeds $1.25 trillion per year.^3^These results can be effective for managers to better understand the safety culture^4^.

There is a rise in the number of occupational accidents in all the World health organization (WHO) regions except for the HIGH and EURO regions. Asia has the highest number of fatalities among the 5 regions. The rate of fatal occupational accidents is 12.7 in 100,000 and 16.6 in 100,000 workers in Asia and Africa, respectively. Europe has the lowest fatality rate among the 5 regions, with a rate of 3.61 in 100, 000 persons^5^.

Despite a 5% increase in employment in the HIGH region, a 6% reduction in occupational accidents has been reported. In EURO, occupational accidents have decreased by 3% with a 2% increase in employment. However, in WPRO and SEARO, although employment has increased by 2-3%, the mortality due to occupational accidents has increased by about 8-9% ^6, 7^.

In Iran, few studies have evaluated occupational accidents. According to one study in 533 registered occupational accidents in 2008, the most common cause was trapping which mostly occurred in the morning shift. The highest incidence of occupational injuries was seen in metal and construction industries. Moreover, 10.8% and 9.3% of the cases resulted in death and permanent total impairment, respectively^8^.

In another descriptive study conducted in an industrial city of Iran (Yazd), falling down accounted for 20.8% of all causes, and hands were the most injured body part^9^.

Bakhtiari et al conducted the largest and most extensive study of occupational accidents in Iran. According to the results of this study that was done during 5 years (2001- 2005), 98.7% of cases were men and total incidence rate was 0.3%. About 3% -%5 of occupational accidents resulted in disability and death annually^10^.

Basically, data collection and logical analysis is the first step in planning and preventing occupational accidents. Since no study has investigated the trend of occupational accidents in Iran in the recent years, and the present research is the largest and most comprehensive study about the occupational accidents in Iran.

The aim of this study was to examine the trend of occupational accidents during 10 years (2007-2016) and to find the association of accident outcome with demographic and occupational characteristics.

## Methods

This cross-sectional study was done based on the data collected by the Iranian Social Security Organization (ISSO). Information on occupational accidents is recorded annually based on the report of the work inspector in the ISSO Electronic System.

According to the ISSO regulations, an occupational accident is defined as a sudden and unexpected accident in the workplace resulting in physical or mental injuries. An occupational accident is considered an accident that occurs in the work place, on the way home from work or vice versa, or when the employee is away on a mission by the employer^11^. Employers must report occupational accidents to the ISSO within three working days after its occurrence, and then the social security inspectors visit and evaluate the workplace and gather the required information about the incident through an interview with the person involved in the incident and his/her colleagues^11^.

In Iran, Similar to many countries, the government has established a social security organization to provide compensation for work-related diseases and accidents. The medical and rehabilitation costs as well as partial and full compensation for salary loss are covered by the ISSO.

All injured workers insured by the ISSO during 2007-2016 were included in the study. According to the national guidelines of ISSO and based on the result of occupational accident the person may be total, partial and minor disabled.

Total disability is a condition in which an individual is no longer able to work at all due to an occupational injuries. Workers with 66% disability or more, are called total disabled.

Partial disability is defined as any type of disability in which the workers is unable to perform at full physical capacity.  Workers with between 33% and 66% disability due to occupational accidents, are called partial disabled.

Minor disability (wage compensation) refers to the funds, which are paid during minor occupational injury or temporary unemployment^11^.

Demographic data such as age, sex, and marital status as well as the occupational accident characteristics including accident sector, cause, type, body part, location, time, month, and outcome were investigated. We defined three occupational sectors including a. agricultural sector (farming, fishing, and forestry), b. industrial sector (mining, manufacturing, energy production, and construction), and c. service sector (office workers, public service, transfer, business). Workers who had an occupational accident between 2007 and 2016 were included in our study. Subjects, who suffered an accident rather than work, were excluded from the study. A The occupational incidence rate was calculated as the number of cases suffering from occupational injury during the reference period divided by the total number of workers in the reference group during the reference period multiplied by 1000.

The severity rate is defined as a number of work days lost multiplied by 100 full-time equivalent workers working 40 hours/week, 50 weeks/year (200,000) divided by total hours worked by all employees during calendar year.

Data were analyzed using the SPSS software version 20. P values equal or less than 0.05 was considered as significant. ANOVA, chi square, and multinomial logistic regression were applied for data analysis.

This study was approved by the Ethics Committee of Tehran University of Medical Sciences, and the data were anonymous.

## Results

The data of 207604 workers suffering from occupational accidents during a 10-year period were collected. The mean (SD) age of the injured workers was 32.97 (9.15) years. Male subjects had a higher frequency than female subjects. More than two-thirds of the injured subjects were married. The frequency of occupational accidents was higher in the morning shift.

In terms of the accident location, 78% of the injuries occurred inside the workplace. The most common cause of accidents was incaution and lack of attention (60%). As for the accident type, collision, hitting, and trapping had a higher frequency than other accident types. The results showed that the upper extremity (36.2%), lower extremity (34%), and spine (18.1%) were the most affected body parts in the accidents. Moreover, 0.4% to 0.6% of the occupational accidents resulted in death during 2007-2016 (Table 1).

**Table 1:** Occupational accident characteristics

**Variables**	**Number**	**Percent**
**Accident location**		
Inside workplace	195,908	94.4
Outside workplace	9,595	5.6
**Accident cause - unsafe condition**		
Equipment malfunction	8,070	3.7
Unprotected safeguards	11,850	5.4
Unsafe work environment	2,593	1.2
Improper management and training	4,197	2.0
**Accident cause - unsafe act**		
Incaution and lack of attention	1,301,754	60.0
Failure to use protective equipment	4,780	2.2
Adopt safety rules	11,604	5.3
Others	44,136	20.2
**Accident type**		
Collision, hit and trapping	131,935	59.3
Falling	40,738	18.3
Burning	6,231	2.8
Acute accidents	31,500	14.2
Electrical shock	1,595	0.7
Others	8,709	3.9
Poisoning	1,306	0.6
**Body part**		
Head& neck	11,497	5.2
upper extremity	80,439	36.1
lower extremity	75,663	34.0
spine	40,297	18.1
Trunk	734	0.3
Abdomen & pelvic	421	0.2
whole body	2,625	1.2
Others	10,836	4.9
**Accident outcome**		
Death	1,079	0.5
Total disability	2,050	1.0
Incomplete disability	3,401	1.7
Minor disability	10,237	4.9
Recovery	190,837	91.9

**Figure 1:**
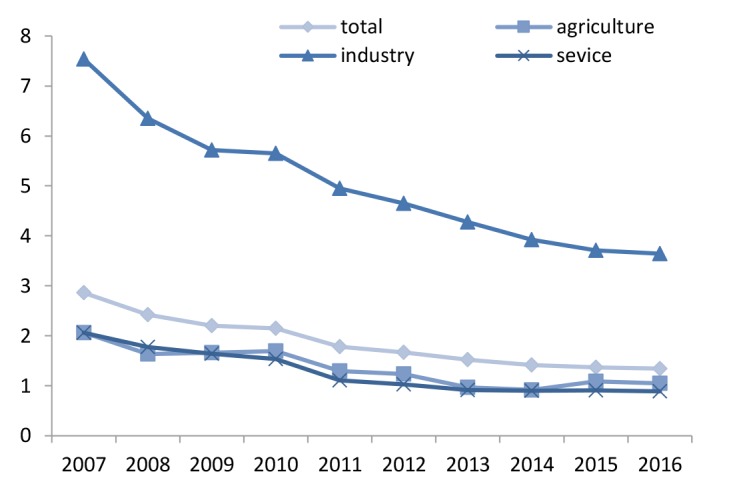
occupational incident rate (total and different sectors)

Figure 1 represents a different data with respect to incidence rate of work related injuries among insured workers by ISSO during the studied years. The injury rate decreased in 2016 (1.34 per 1,000 workers) in comparison with the previous years.

**Figure 2:**
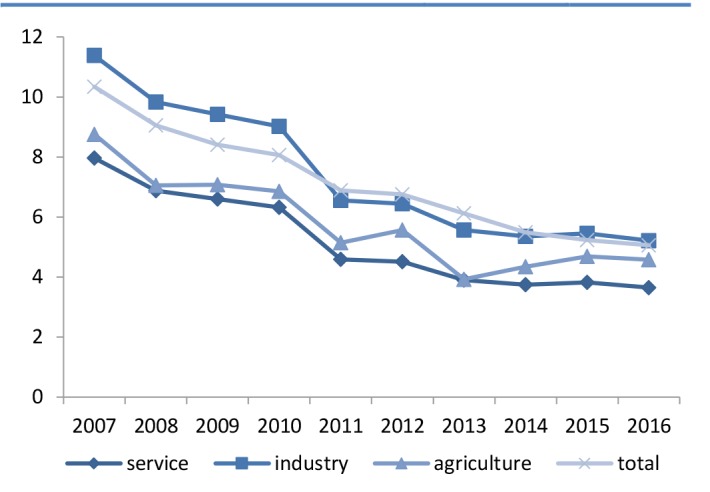
occupational severity rate (total and different sectors)

Figure 2 shows the severity rate for every 1000 people among workers during 2007–20016. The highest severity rate was seen in the industrial sector during all years.

**Figure 3:**
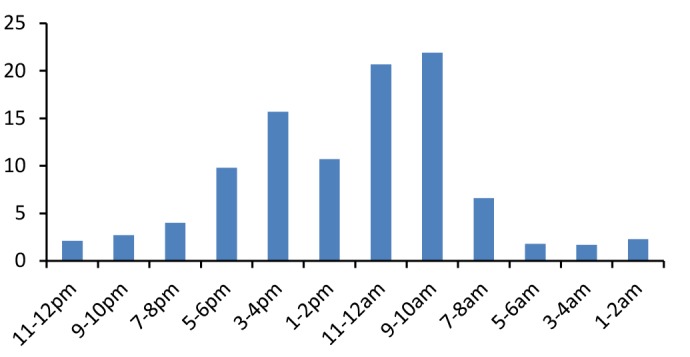
frequency of accidents at different times of the day

**Figure 4:**
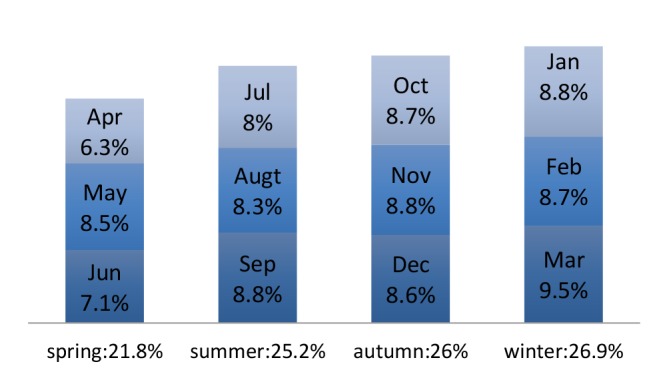
Frequency distribution of accident based on season and month

As shown in figure 3 the most of the accidents occurred during 9-10 am. Work related injuries were more likely reported in the winter (26.9%) and autumn (26%); therefore, the lowest rate of accidents was seen in the first half of the each year. (Figure 4)

**Figure 5:**
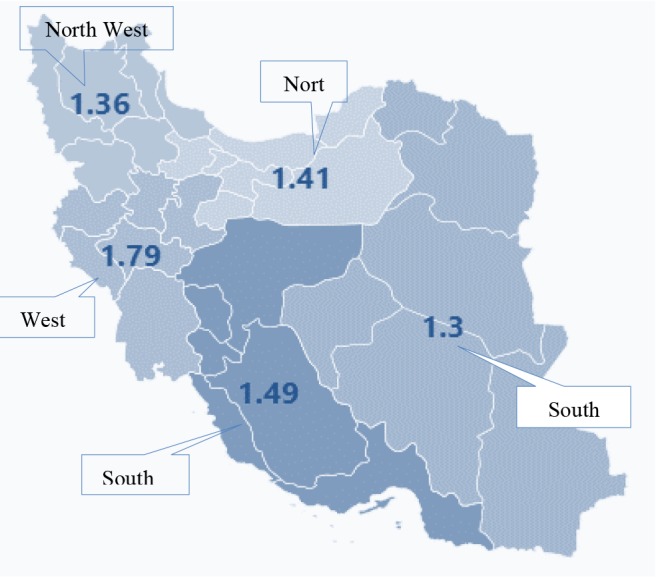
Injury rate per 1000 worker in 5 geographical regions

Considering job-related injuries, it was found that the west region had the most (1.79 per 1000 worker) and the southeast of Iran (1.3 per 1000 worker) had the least rates injury rate. (Figure 5)

The fatality ratios were 1.37 (per100, 000 workers) and 0.85 (per 100,000 workers) in 2007 and 2016, respectively.

According to Table 2, age, gender, marital status, activity, season, work place, and shift work were associated with the accident outcome in univariate analysis.

**Table 2:** Relationship between demographic and occupational characteristics with accident outcome

**Variables**	**Total**	**Death**	**Total** **disabil ity**	**Partial** **disability**	**Minor** **disability**	**C omplete** **recovery**	***P*** **value**
**Sex**							0.001
Male	203,322	1074	1,997	3,317	9,967	186,967	
Female	4,281	5	53	84	270	3,869	
**Marital status**							0.001
Single	46,874	133	468	767	2,362	43,144	
Married	160,730	946	1,582	2,634	7,875	147,693	
**Activity**							0.001
Agriculture	5,279	43	78	143	370	4,645	
Industry	149,083	605	1,324	2,333	7,577	137,244	
Service	53,242	431	648	925	2,290	48,948	
**Season**							0.001
Spring	45,331	251	504	891	2,294	41,391	
Summer	52,329	272	527	841	2,479	48,210	
Autumn	54,043	255	485	833	2,744	497,26)	
Winter	55,901	301	534	836	2,720	51,510	
**Workplace accident**							0.001
Inside	195,908	817	1,720	2,981	9,426	180,964	
Outside	11,696	262	330	420	811	9,873	
**Shift work**							0.028
Fix	193,392	720	1,398	2,334	6,744	127,318	
Rotatory	14,212	359	652	1,067	3,493	63,519	

The same results of univariate analysis were observed in multinomial logistic regression except for shift work (Table 3).

**Table 3:** Relationship between different factors and accidents with outcomes: adjusted odds ratio and 95% confidence interval with multinomial logistic regression (the reference category is recovery)

**Variables**	**Recovery**	**Death**	**Total disability**	**Partial disability**	**Minor disability**
Age	1	1.03 (1.02, 1.03)	1.02 (1.02, 1.03)	1.02 (1.02, 1.03)	1.01 (1.01, 1.02)
Sex	1	0.25 (0.10, 0.59)	1.18 (0.89, 1.60)	1.14 (0.98, 1.43)	1.25 (1.10, 1.40)
Marital status	1	1.34 (1.09, 1.63)	0.78 (0.69, 0.87)	0.78 (0.70, 0.86)	0.85 (0.80, 0.90)
Shift work	1	1.01 (0.91, 1.10)	0.95 (0.88, 1.02)	0.95 (0.89, 1.00)	1.01 (0.98, 1.04)
Job category	1	1.29 (1.15, 1.46)	1.05 (0.97, 1.20)	0.89 (0.83, 0.96)	0.79 (0.75, 0.82)
Season	1	0.99 (0.94, 1.05)	0.95 (0.91, 0.99)	0.92 (0.89, 0.95)	0.99 (0.98, 1.01)
Accident location	1	4.88 (0.42, 5.64)	3.31 (2.90, 3.70)	2.52 (2.26, 2.80)	1.63 (1.51, 1.76)

## Discussion

This study was the first study that used all accidents recorded by the ISSO. The results showed that occupational accidents had a decreasing trend during 10 years of the study from 2.95 per 1000 workers in 2007 to 1.46 per 1000 workers in 2016.

According to the ILO, approximately 313 million occupational accidents occur annually, and the 6400 workers dies every day. According to our data, the frequency of occupational accidents was 24152 and 18523 in 2007 and 2017 respectively, of which 0.4-0.6% were fatal accidents. Although most industries and high risk occupations are covered by the insurance system, the responsibility for health and safety at work is divided between the Labor Organization and ISSO, otherwise the insurance coverage in our working population is about 45-50% during different years. Therefore, it sees the rate of work-related occupational accidents is underestimated.

However, the reduced rate occupational accidents despite increased insurance coverage of the working population during these years could be due to development of the occupational safety and health system.

The industrial sector had the highest rate of recorded occupational accidents, but the frequency of fatal occupational accidents was two times higher in the agricultural sector. In low- and middle-income countries of the South-East Asia Region (SEARO), the agricultural sector has the rate of 17 deaths per 1000000 workers, while the industrial sector has the fatality rate of 13.4 per 100000 workers.^3^In several European countries and the United States, the rate of fatal accidents in the agricultural sector is more than the average of other industries^12^.For example, the fatal accident rate of the agricultural industry in the United States is the highest fatality rate compared to any industry (23.2 per 100,000 workers)^13^.

According to the ILO, agriculture, construction, mining, and ship-breaking are known as the most hazardous occupations .The variability in working conditions, including sudden climate change, is the characteristic of most agricultural activities.

Iran as a vast country has regions with different climates even at a specific period of time. According to the results of this study, the maximum and minimum number of occupational accidents was seen in the winter and spring, respectively. The weather in the winter is associated with various hazards, including slippery surfaces, poor winter driving conditions, cold stress due to extremely cold temperatures, and electric shock from the use of power equipment. These conditions may result in an increase in occupational accidents. A higher and lower frequency of accidents was found in March and April, respectively. The official Iranian calendar is based on the Solar year and March is the end of the Iranian Year. At-the-end-of-year (March) injury rates are pronounced because of higher workload and a lower frequency of accidents in April is expected because of public holidays (14 days) in this month.

Furthermore, based on our results, unsafe acts (67.5%) had the most important role in occupational accidents. According to Herbert William Heinrich who is an American industrial safety pioneer, "unsafe acts are responsible for 88% of accidents, whether the cause of 10% of accidents is unsafe conditions and the cause of 2% of accidents is unforeseen factors".

According of the results of two studies in Iranian industrial companies, 24.5% and 26.7% of workers’ acts were unsafe, respectively^14, 15^. Researchers have shown a significant association between unsafe acts and level of education. Therefore, implementing behavior-based training programs is recommended to reduce unsafe actions.^6^.

Collision, hitting, and trapping were the most common causes of occupational accidents in our study. The main mechanical risks include crushing, shearing, cutting, falling, trapping, and hitting, which are caused by mechanical elements such as tools, parts, solid material, etc. The consequences of these injuries are often severe, such as crushed limbs, amputation, and death. In an analysis of industrial accidents carried out in Poland during 2005-2011, it was shown that the basic causes of accidents were trapping by moving elements of the machinery, crushing, hitting and grasping^17^.

The body parts most vulnerable to injury were the upper and lower extremities, respectively. According to the United States Department of Labor in 2014, injuries to the upper extremities occurred in 346,170 cases, or in 32 cases per 10,000 full-time workers. Hands accounted for 40% of those cases^18^.Prevention of hand injuries requires identification of modifiable risk factors such as the use of personal protective equipment (gloves), fatigue and use of unusual tools^19^.

The results showed that the probability of severe consequences increased with an increase in the worker’s age. This finding is consistent with the results of other studies indicating that the percentage of fatality is higher in older workers^20^.The reason may be that some safety risks could be due to reduced physiological function as a result of ageing such as age-related hearing loss^21^. It is concluded that injury prevention should address improving relevant lifestyle factors, especially in older worker^22^. Moreover, reassignment of older workers to low-risk exposures and tasks could be effective in reducing this rate.

According to our results, the mortality rate was higher in men than women. In 2016, 4,803 and 387 deaths occurred due to occupational injuries in male and female workers in the United States, respectively^23^. According to Hoskins A.B study, women suffer less fatal and nonfatal injuries at work than men ^24^. The reason may be that women are employed in low risk jobs, causing differences in how they are injured at work. The fatality rate was also higher in married subjects, may be because married people with children become the most averse to risk. Some studies have shown that occupational accidents occur more frequently in married workers compared to single workers^20, 25^.The high rate of accidents in married workers could be due to competing responsibilities at work and at home that may lead to more unsafe acts.

Fatal occupational accidents were statistically significant outside the work station, perhaps as a result of unexpected and unforeseen events. Also, in addition to accidents inside the workplace, an injury occurring on the way from home to work or vice versa is known as an occupational accident; therefore, awareness of one’s surroundings and practicing caution at all times will minimize the potential for an incident to occur.

Many organizational safety programs typically focus on safety and training needs of the workplace itself or the hazards faced by subjects. One common concern to minimize the risks is to provide safety standards and training for outside the workplace.

There are many theories for occurrence of occupational accidents. The causes of occupational accidents are different and depend on many factors such as demographic and occupational characteristics, organizational culture, psychosocial and economic factors, etc.

Ultimately, rather than disability many work days lost. However, many developing countries often lack the resources to maintain an effective safety management system. Furthermore, there is insufficient supervision on the implementation of safety at work in our country, but we ought to remember that safety must be a top priority at all times.

The data of our study were obtained from ISSO records, therefore the real statistics could be underestimated since it is possible that not all occupational accidents are reported by employers. The cross-sectional nature of our study prevents concluding a causal association between related factors. However, it is recommended that more detailed information be collected on occupational injuries to evaluate other risk factors.

## Conclusion

The current survey is truly the most comprehensive and representative study of occupational accidents in Iran in general. Moreover, the probability of a recall bias was minimized through using the ISSO database, which must be completed by the employer within 3 days after the occupational accident. The findings of this study add to the growing body of knowledge showing the importance of some variables in the accidents outcomes. The results underline the importance of identifying the causes of injuries and working conditions and implementing obligations to develop protection programs and safety, which must be ensured and supported by the employer.

## Acknowledgements

The authors would like to thank the educational and statistic departments of the ISSO for their contribution to this study.

## Conflict of interest

The authors declare that there is no conflict of interests to declare.

## Funding

None.

## Highlights

- Occupational accident statistics over the recent 10 years shows a slight decrease in Iran.
- The greatest reason of accidents was incurious activities and behaviors.
- Accidents in the industrial sector has a substantially higher frequency and severity rate than other sectors.
- Old age, male gender and married subjects were found to be associated with more severe outcomes.
